# Indents

**Published:** 1904-08-15

**Authors:** 


### INDENTS.

☞ **Brief Articles of Technical or Scientific Value will receive notice and comment. Contributions invited.**

**Abortive Treatment of Boils.** Gallois (*Therapie der Gegenwart*) advises for this purpose that the boil should be painted with a concentrated solution of iodin in acetone. This solvent takes up four times as much iodin as alcohol, and its use on a boil is followed by the formation of a brown scab, under which the inflammation subsides. The solution should be kept for some weeks before it is used.—*Digest.*

**Editor and Doctor.** If an editor makes a mistake, he has to apologize for it, but if a doctor makes one he buries it. If the editor makes one there is a lawsuit, swearing and the smell of sulphur, but if the doctor makes one there is a funeral, cut flowers and a smell of varnish. The doctor can use a word a yard long without knowing what it means, but if the editor uses it he has to spell it. If the doctor goes to see another man's wife he charges for the visit, but if the editor goes to see another man's wife he gets a charge of buckshot. When a doctor gets drunk it's a case of "overcome by heat," and if he dies it is heart trouble. When an editor gets drunk it's a case of too much booze, and if he dies it's a case of delirium tremens. Any old medical college can make a doctor. You can't make an editor. He has to be born.—*Factotum.*

**Eau Dentifrice.** Dieterich recommends the following formula for a mouth and tooth-wash:

| | |
|---|---|
| Rhatany | 100 parts |
| Chinese cinnamon | 5 parts |
| Distilled water | 80 parts |
| Alcohol, 90 percent | 20 parts |
| Salicylic acid | 1 part |

Pulverize the rhatany and cinnamon, mix the ingredients, and for each quart of liquid add ten drops of peppermint oil, two drops of cloves, and one drop attar of Ylang Ylang, dissolving them in the alcohol before mixing. Let macerate for one week and filter.

Cracan gives another good formula, as follows:

| | |
|---|---|
| Star anise seed in powder | 30 parts |
| Rectified spirit of wine | 400 parts |
| Oil of star anise | 5 parts |
| Oil of peppermint | 5 parts |

Mix. Color with alkanet root.—*Pacific Drug Review.*

**Sugar and Dental Caries.** There can be no doubt that a mistake has been made in the past regarding all sugars as similar, and that, in the future, in their relation to caries, they must be looked upon as different substances transformable, to some extent, into each other—just as peptone, being a proteid, is a changed form of other proteids, though possessing many different properties.

Experimentally, I have so far some reason to believe I am correct in saying that the main cause of caries in man throughout all time has been due to the introduction of the more easily fermentable carbohydrates into his diet, and the more recent increase due to the introduction and widely-spread use of artificial glucose. If it be so, then at least we may have reason to believe that an almost complete prevention of caries is not a hopeless dream, but one well within a reasonable range of possibility.—S. Calyer, in July, 1904, *Record.*

**Hydronaphthol In Dental Therapeutics.** Hydronaphthol is too well known to need any description now. Its first and greatest claim is that it is a powerful antiseptic and yet is non-toxic and non-irritant, and—what to us, as dentists, is very important—the odor is not disagreeable. For convenience in using I keep hydronaphthol mixed with cement powder in the different proportions I generally use. I mix by weight, using one part hydronaphthol to five of powder, also one part to ten, one to twenty, one to fifty, and one to one hundred. With these different proportions prepared and put in bottles, I can use them as readily as the plain cement powder. In a cavity so deep that I know I am unable to remove all infected dentin, I use the proportion one to one, or one to five, to be covered with one to one hundred, or with clear cement or amalgam. If I want to put a non-conductor under gold, I use one to twenty; under an inlay in a deep cavity, I use one to twenty or thirty; for filling a pulp chamber, one to twenty; for setting an inlay, one to one hundred.—E. A. Royce, *Review*.

**Immunity of the Tissues of the Dental Cavity to Auto-infection.** Dr. R. H. M. Dawbarn stated as his opinion that the explanation of the well-known immunity of the tissues of the oral cavity to auto-infection lies in the fact that from birth until death the region under discussion is vaccinated, so to speak, with ptomains and toxins, the products of the life activities and death decompositions of the germs normal and native to that region; and that because of such vaccinations, the outcome of the numerous slight lesions so frequently occurring, the flesh thereabouts is in some way made better able to combat and overcome any ill results when greater wounds are received. Then, too, it is a fact worth noting that each healthy individual's saliva is not dangerous to himself, even elsewhere than the mouth, wounds, or fractures, though this secretion would be perilous to another individual. Probably the reason for this is the one already mentioned—a continual vaccination. We have all seen wounds upon the hands of street urchins sucked and washed clean by their spittle, just as a dog cleanses himself, with no ill result.—*Digest*.

**Children Patients.** In the treatment of children make it a point first to be truthful; never an untruth under any circumstances. A great point is to have the patients, especially the small ones, rid themselves of the idea that they are about to be killed. How many times do we see patients holding themselves under a strain, expecting every moment to be called upon to endure excruciating pain? After the patient and myself are somewhat acquainted, I say to him: "Now I'm not going to hurt you for the present; before I begin to hurt you, I'll let you know, and you can get ready." I then proceed with the preparation of the cavity; perhaps a chisel is used to break down the enamel—much noise, but little pain. If I see the patient is still under a strain and acts as though every minute is to be his last, I stop and say: "Did that hurt you? No! I thought not." It puts him more at his ease. If the patient is a child, I say: "Did I ever tell you an untruth? Now I mean just what I say; it is *not* going to hurt. If you don't believe this, I shall think you have no confidence in me." "But doctor, it makes such a noise!" "Yes, so it does. But did you ever hear the story of the old farmer who wouldn't buy a lightning rod? He wasn't afraid of the lightning, but if the agent had any *thunder* rods, he would take a half a dozen!"—W. W. Belcher, in *Office and Laboratory*.

**Appendicitis and the Teeth.** " 'Bolting' the food is one of the most pernicious causes of appendicitis," says Dr. Russell Combe, in a late number of the *Lancet*.

Young men suffer from the disease more frequently than their elders. In fact, nearly two-thirds of the cases treated in the past sixteen years were persons under thirty years of age. Bad teeth add to the danger of appendicitis, and frozen and tinned foods increase the chances of contracting it.

That cases of appendicitis have become more frequent during the last ten years, Dr. Russell Combe says there is no doubt. "What then, is the cause of this increased frequency?" asks Dr. Combe.

"Eighty percent of cases occur under thirty years of age;

73 percent of cases occur in males; therefore, 60 percent of cases (nearly two-thirds) occur in males under thirty years of age. It has been stated that whatever its cause, it must be one that affects all classes of the community, but I would point out that these figures suggest a cause especially affecting young males.

"We may reflect that it is during the past sixteen or eighteen years that appendicitis has assumed its present importance, and I believe that dental surgeons will uphold the view that the same period has shown the gravest deterioration in the fitness of the molar teeth for mastication. Admit this point, and, further, remember that young men are especially the class who are prone to hurry their meals and often have to pick them up irregularly as best they may.—*Record.*

**Antrum Empyema and Treatment.** It is evident to any one who scans the literature on the treatment of empyema of the maxilliary sinus, that there is a wide difference of opinion as to what is the best surgical method of procedure. The old method introduced by Hunter, Cooper and Astley, which is the opening of the cavity of the antrum through the alveolar or canine fossa, or whether the more modern method of perforating the lateral wall of the middle of inferior nasal meatus. The object in each case is drainage. We have in all cases followed the route through the alveolar arch. We use chloroform in all cases. Every attention should be given to asepsis; the field of operation should be cleansed with hydrogen peroxide, and followed by a saturated solution of boric acid. To obtain perfect drainage the lowest point of this bony shell should be tapped. If the second bicuspid or the first and second molar are absent or diseased, these points of entrance should be selected and a small dental twist drill directed upward, inward and slightly backward until the bone has been perforated, pus or serum follows the removal of the drill. The opening should be dilated to the size of a lead pencil, and the surrounding tissues thoroughly removed from the margins of the opening. Perfect drainage having been secured, there are few cases that will demand anything more. Cleanliness ranks first of all agents in securing good results. Plugs, tents and dressings are often factors in keeping up irritation and holding infection.

The writer is convinced that if the average dentist would make a systematic examination of every patient, empyema of the antrum would be diagnosed early, that drainage would be rewarded with speedy recovery.—Dr. McNerthney, in *Dental Summary.*

**Co-education in Dental Colleges.** President Angell, of the University of Michigan, in one of his annual reports employs the following strong language: "After our twenty-nine years' experience in co-education we have become so accustomed to see women take up *any* kind of university work, carry it on successfully, graduate in good health, *cause no embarrassment in the administration* of the institution, and *awaken no special solicitude in the minds of their friends or teachers*, that many of the theoretical discussions of co-education by those who have not had opportunity to examine it thoroughly read strangely to us here on the ground."

As final evidence on this point, hear our own Professor Peirce. Writing to us recently, he said: "I am now and always have been in favor of co-education. It is now nearly thirty years since I admitted women to our dental school on the same footing as men. When it was first done I was much amused one day to have a committee of students present themselves in my office on what they considered important business. 'Well,' I said, 'gentlemen, what is the business?' They said they came to protest against women being in the class. I replied, 'Well, gentlemen, I believe you have not been asked. When I want your opinion on the subject, I will send for you, but I want to tell you now that the women are here to stay.' This ended the whole contest, and we have had women ever since. They first have a refining influence; there is less boisterousness, vulgarity and profanity; less spitting and other unseemly conduct. This, of course, has reference to the laboratory, but in the lecture-room also they have a modifying influence, so that conduct is always improved by the presence of the female sex. I have no private opinion on this subject. I am unconditionally for co-education. The rose always adorns the bush even though it is thorny."—Dr. Chapple in *Digest.*

**A Camphenol-like Antiseptic.** The antiseptic which I wish to bring before your notice is a simple one; it is a modification of camphenol or camphophenique. To prepare it, take two fluid ounces of pure liquid carbolic acid and equal parts camphor, menthol and thymol. Place the latter substances in a colored three-ounce bottle, pour the acid over and shake. It immediately becomes milky. Complete solution takes two or three days. More menthol, camphor and thymol should be added till acid is saturated. Decant clear solution and it is ready for use. It should be kept in a brown or dark blue bottle with a well-fitting glass stopper. Indications: A pulpless tooth with chronic abscess; gain access to canal, syringe out with H2 O2, three percent, treat with sodium dioxide, dry canal and flood with antiseptic, forcing it into the abscess sac and out through sinus, if one be present. The apex is now sealed, and subsequent treatment is done through the sinus, one drop of the agent being placed in the sac by means of a hypodermic syringe. Sometimes one, but generally two or three sittings are necessary to bring about a cure; it is not good treatment to hurry with a chronic abscess. It is useful, used in full strength, for wiping out cavities prior to filling. For pulpitis it is valuable. A small pledget of cotton-wool moistened with it, placed over the pulp and covered with a light dressing, inserted without pressure, will give immediate relief. Four minims in half a tumbler of warm water makes a good mouth-wash, leaving behind an agreeable sensation of cleanliness. It is, however, not recommended as a mouth-wash for constant use, but mainly after extractions or operations in the mouth. It is an efficient agent for the relief of pain after the extraction of teeth with periodontitis. I regard it as next in value as an all-around antiseptic and germicide to hydrogen peroxide. It is very simple to make; the addition of saccharine gr. v. to every ounce makes it more acceptable to patients as a mouth-wash.—Dr. Bernard Bennette, *Record*.

**Sugar and Toothache.** The sugar plantations in Natal are worked by coolie labor, the coolies being imported from India for the purpose. Each plantation has its coolie quarter—a little world in itself. It has also its own private hospital, and a medical man from the nearest village attends daily. The principal ailment these people suffer from is toothache. The young cane is very luscious; it is also very nourishing. The food the coolie lives on is not rich in health-sustaining ingredients; it consists principally of rice, oil, dholl, and ghee, so the people make up the deficiency by chewing cane continuously. The saccharine gets in its fine work, and the result is almost universal toothache. The ailment, in fact, is so common that if a man should plead it as an excuse for absenting himself from work, he would be put down at once as a "shirker." The women and children are also very partial to the cane, and the children's teeth are a study in themselves. The climate here, on the coast line, is very enervating, and this also has an injurious effect. The coolie does not believe in having his teeth extracted. In this case it would avail him little to do so, for as long as he had one molar remaining, he keeps on at his pernicious habit, so he suffers in silence, though at times his sufferings lead him to an over-indulgence in the white rum manufactured on the estates. The people are well aware of the effects of indulging in this craving. New batches of arrivals are always warned by the overseers, but the warning has little effect. It is warm working in the sun, the rice water in the buckets is at summer heat, the cane is cool, and the juice invigorates, so the warning is unheeded. "I can tell you how long a man has been on an estate by his teeth," said an overseer to the writer; "that is to say," he corrected himself—"any time up to two years; after two years the teeth get into such a state that they can deteriorate no farther, as far as outward appearances go, at any rate." Europeans on the coast line also suffer from toothache; this, however, is charged to the climate, and the deterioration in their case is very slow in comparison to what it is in the case of the coolies. The coolie saves all the money he earns, and on completion of his indenture is given a passage back to India. He is now a man of substance, in his own eyes—that is to say, he has a capital of from £15 to £20, but many a one would forfeit this if by so doing he could rid himself of the demon ache with which he is tortured.—D. S. Ricard, in *Record*.

**Filling the Matrix.** The proper color having been selected, a portion of it is placed upon a clean glass or porcelain slab, water added to it and the mass thoroughly spatulated. The excess of water, if any be present, is then taken up with a linen napkin or bibulous paper. The matrix should be grasped slightly but firmly on some portion of the surplus with a pair of pliers that can be closed and set. This insures against the dropping of the matrix. A little of the moist body is taken upon the point of a spatula and dropped on the floor of the matrix. A serrated instrument is drawn over the pliers, or they may be tapped slightly to settle the particles of body closely together. In doing this, the moisture is driven to the surface of the mass. It can be removed by touching it lightly with a small roll of bibulous paper and the process repeated until the procelain is settled into a dense homogeneous mass.

Only a small amount of body should be applied for the first baking, and this should not be allowed to lie against the matrix walls to any extent, or warpage of the latter, due to contraction of the porcelain, may result.

The first baking should be continued until the porcelain is fully glazed, the temperature being carried a trifle higher possibly than will be required for subsequent bakings. This induces the maximum shrinkage in the inlay at this point and gives an unchangeable base on which to apply additional layers of body.

The second application of body should be made in a similar manner to the first, being careful to condense it thoroughly by vibration. This time it should be built up against the walls of the matrix to perhaps half the depth of the cavity when large, and nearly full when small.

The second baking is then made and the piece well glazed. At this time some inlay workers prefer to place the matrix with the partially-completed inlay in the cavity to ascertain if warpage has occurred, and if so, to correct it by additional burnishing.

The third application is now made, the matrix filled slightly fuller than the required contour, and the paste around the margins trimmed exactly to the angles, allowing no surplus to extend over the margin at any point.

Upon baking the third time, should the margins be below the required level, additional body should be added and the case returned to the furnace. The last baking should produce an even uniform glaze on the inlay surface.—Dr. Prothero, in *Northwestern*.

